# Feasibility of Using Electronic Health Records for Cascade Monitoring and Cost Estimates in Implementation Science Studies in the Adolescent Trials Network for HIV/AIDS Interventions

**DOI:** 10.2196/25483

**Published:** 2022-04-25

**Authors:** Tyra Dark, Kit N Simpson, Sitaji Gurung, Amy L Pennar, Marshall Chew, Sylvie Naar

**Affiliations:** 1 College of Medicine Florida State University Tallahassee, FL United States; 2 College of Health Professions Medical University of South Carolina Charleston, SC United States; 3 New York City College of Technology City University of New York Brooklyn, NY United States

**Keywords:** EHR, HIV, youth, viral load, antiretroviral therapy, treatment cascade

## Abstract

**Background:**

One of the most difficult areas in the fight against HIV/AIDS is reaching out to youth aged 13 to 24 years. The proportion of youth living with HIV/AIDS on antiretroviral therapy (ART) and who are virally undetectable is low, highlighting significant challenges for reaching the Joint United Nations Program on HIV targets.

**Objective:**

This study aimed to assess the feasibility of obtaining key clinical indicators and monitoring treatment, viral suppression, and retention components of the youth HIV treatment cascade in Adolescent Trials Network for HIV/AIDS Interventions clinics using electronic health record (EHR) downloads and to provide baseline characteristics for the study participants.

**Methods:**

EHR data were systematically obtained from multiple clinical sites and used to meaningfully capture clinical characteristics, initiation of antiretrovirals, and retention in care, which are part of the Centers for Disease Control and Prevention’s 4 continuum of care measures. In addition, this study used standard cost values attached to Current Procedural Terminology codes to estimate the cost per visit.

**Results:**

Only 2 of the 4 Centers for Disease Control and Prevention treatment cascade measures were assessed using routine EHR data. EHR data are not adequate for monitoring HIV testing or linkage to care because denominator data are not available. However, the data work well for measuring ART initiation and adequately for retention in care. The sites were broadly able to provide information for the required data. However, in most cases, these data are insufficient for identifying patterns of missed appointments because such misses are not captured in the EHR system. Sites with good access to data management resources can operate more efficiently for cascade monitoring study purposes.

**Conclusions:**

Data other than EHRs are needed to measure HIV testing and linkage to youth care. EHR data are useful for measuring ART initiation and work moderately well for measuring retention in care. Site data management resources should be part of the selection process when looking for site partners for clinical studies that plan to use EHR data. Study planners should determine the feasibility of additional funding for organizations in need of additional information technology or data management resources.

## Introduction

### Background

We have made substantial progress in preventing HIV infection in the United States during the past decade, and the federal government has recently released a national strategic plan to end the HIV epidemic in the United States by 2030 [1]. However, one of the most difficult areas in the fight against the HIV and AIDS epidemics is reaching youth. In the United States, in 2016, approximately 21% of the 39,782 new HIV diagnoses occurred among youth aged 13 to 24 years [2]. We will not be able to end the HIV epidemic in the United States unless we reach this age group. However, preventing new HIV-positive diagnoses will require a 2-pronged attack, because prevention must reach youth at risk of acquiring HIV and treatment must reach those already living with HIV. The latter is an often-neglected group [3]; however, we cannot conquer the HIV epidemic in the United States without solving the youth *treatment cascade* (ie, HIV testing and diagnosis, linkage to care, and viral suppression) problem.

National-level strategies specify goals related to early HIV diagnosis and effective care. The Centers for Disease Control and Prevention (CDC) HIV care continuum identifies a dynamic series of steps from the time a person receives a diagnosis of HIV through the successful treatment of their infection with HIV medications [4]. The HIV care continuum consists of several steps that are required to achieve viral suppression. Specifically, CDC tracks the following: (1) diagnosed—receives a diagnosis of HIV, (2) linked to care—visited an HIV health care provider within 1 month after learning they were HIV positive, (3) received or were retained in care—received medical care for HIV infection, and (4) viral suppression—their HIV viral load (VL) was at a very low level. Although the HIV care continuum is often presented as a static framework, individuals who are HIV positive often exit and re-enter the continuum at varying steps [5]. Although relatively straightforward conceptually, programmatic implementation to systematically monitor the HIV treatment cascade is quite challenging [6]. Despite the fact that studies have used study coordinator data entry of cascade variables for consented participants [7], this does not adequately reflect the range of youth at Adolescent Trials Network for HIV/AIDS Interventions (ATN) clinics who may not consent to such trials. To date, no studies have been published on multisite youth cascade variables using electronic health records (EHRs). To that end, Scale It Up (SIU), a collaborative program (U19) within the ATN, aims to bring to practice evidence-based self-management interventions via hybrid implementation trials to positively impact the youth HIV prevention and care cascades.

### Objectives

The Cascade Monitoring (CM) protocol (ATN Protocol 154) was designed to monitor the cascade within the ATN using EHR and provide longitudinal effectiveness outcomes and cost estimates [8]. The first goal of ATN 154 and the purpose of this report are to assess the feasibility of assessing the youth HIV treatment cascade [9] among those linked to care within the ATN using EHR downloads. To this end, this study aims to assess the feasibility of obtaining key clinical indicators and monitoring treatment, viral suppression, and retention components of the youth HIV treatment cascade in ATN clinics using EHR downloads. In addition, this study will use the first data submissions from 10 study sites to estimate the cost per visit and provide baseline study participant characteristics.

## Methods

### Ethics Approval

In compliance with ethical standards, the SIU CM study (ATN 154) was approved by an expedited review process by the single institutional review board of Florida State University (approval IRB00000446). All the procedures performed in this study were in accordance with the ethical standards of the institutional review board.

### Study Sites

Limited EHR data were collected retrospectively, from 10 clinical sites, also known as subject recruitment venues, participating in SIU: (1) Johns Hopkins University, Baltimore, Maryland; (2) University of Alabama at Birmingham, Birmingham, Alabama; (3) SUNY Downstate Medical Center, Brooklyn, New York; (4) Children’s Hospital Los Angeles, Los Angeles, California; (5) St Jude Children’s Research Hospital, Memphis, Tennessee; (6) University of Miami, Miami, Florida; (7) Children’s Hospital of Philadelphia, Philadelphia, Pennsylvania; (8) University of California San Diego, San Diego, California; (9) University of South Florida, Tampa, Florida; and (10) Children’s National Health System, Washington, District of Columbia.

The first data extract requested treatment visits in the full year of 2016 and associated care data for all youth living with HIV/AIDS, aged 15 to 24 years, treated at sites. Subsequently, 1-year data EHR extracts were received from sites annually, with the final year of data uploaded in 2022 for the full year of 2021. Data were requested for a set of variables that were considered invariant for the year 2016 (demographics) and for variables where multiple annual values should be captured to measure the treatment cascade changes. The variables requested included demographics (age, sex, race, and ethnicity), height, weight, International Statistical Classification of Diseases and Related Health Problems–10th Revision (ICD-10) codes, and Current Procedural Terminology (CPT) codes. A complete list of the requested variables has been published elsewhere [8] and is also included in Multimedia Appendix 1.

### Study Clinical Measures

EHR data were systematically obtained from multiple clinical sites and used to meaningfully capture clinical characteristics (including viral suppression), initiation of antiretrovirals (ARVs), and retention in care, which are part of the CDC’s diagnosis-based HIV care continuum measures (Multimedia Appendix 2) [10]. Two of the four CDC treatment cascade measures—retention in care and viral suppression—were assessed using routine EHR data. For the *retention in care* measure, we used the CDC definition of an interval of >190 days (approximately 3 months) between laboratory visits for CD4 or VL testing to indicate less-than-optimal laboratory visit patterns. We were able to obtain laboratory visit dates and use data from the subsequent year to determine whether the participant was retained in care. *Viral suppression* was defined as having a nondetectable baseline VL. Laboratories used by individual study sites have varying lowest detectable limits for VL; therefore, this study records undetectable VL as defined by each study site. Additional measures calculated using EHR data included ARV medication percentage and AIDS status. *Antiretroviral medication (%)* was defined as the percentage of patients with at least one recorded ARV therapy (ART) prescription. The data in the EHR did not allow us to estimate patients’ adherence to ARV because adherence is not documented in the EHR and only issued prescriptions, not prescription fills, are recorded. *AIDS status* was determined by an absolute CD4 T-cell count of <200.

### Feasibility of EHR Data Extraction Process by Study Sites

Multiple EHR platforms are available for the computerized entry of patient medical information. Although a close examination of EHR platforms is important, information on the types of EHR software was not collected because the intent of the CM study was to examine the feasibility of standardized extraction of clinical and cost data of relevance to CM data across multiple EHR systems. Following interviews with study coordinators about the consistency of EHR variable capture, 9 variables were deemed as primary and could be consistently calculated. The following information was provided if available: date of visit, age, sex, height, weight, race, ethnicity, VL, and ICD-10 codes for other diagnoses. Data were requested as comma-delimited files in Excel (Microsoft Corporation). Because the sites have very different access to local technical support for file extraction, the file structure was left to decisions by the sites to ease the workload on the site staff. Data extraction was supported by the provision of an example study data dictionary for each site. This document shows how the variables are optimally defined and delivered. However, the actual extraction files differed for each of the 10 sites in terms of design, organization, variable definition, and completeness. Files had to contain the minimum primary variables, be deidentified with a site-specific patient identifier, and a report provided explaining the reason why any variables were missing. CM study personnel received and approved a variable checklist for each site before the data were approved for uploading. Upon initial receipt of the 2016 data, the data were checked for patterns of variable missingness, congruence with the data request, and the presence of variables for linking relational data by anonymous patient identifiers.

### Data Management

Once the data extract was certified for a site, data were cleaned and transformed into a common data model format. Most of the data cleaning and construction of common data model files were performed using SAS (version 9.4; SAS Institute Inc). Once files for all sites were cleaned and variable values were transformed to fit the common data model for the CM study, we constructed a baseline demographics flat-file with one observation per patient and a set of relational vertical files organized by visit (laboratory test or prescription) date. We constructed separate files for visits, laboratory values, ARV medications, and other medications. The visit file contained all CPT and ICD-10 diagnosis codes and formed the basis for the development of a cost value for each encounter.

### Data Analysis

Costing information was necessary to evaluate whether it was possible to produce visit cost weights using CPT codes from EHR data in lieu of extracting billing and administrative data, which are often difficult to obtain from a clinic. The ability to estimate cost is important for measuring variations in resource use and efficiency in the process of care at clinical sites. These estimated costs will be examined for validity using a time-driven activity-based costing approach once more data are collected. We used a standard costing approach to assign a cost value to each encounter, because EHR data rarely contain cost data. Cost data for individual sites are usually located in a Charge Master file, which is updated as prices at the site change. The Charge Master files are combined into billing costs for a visit using the clinic accounting data system. Thus, EHR data do not contain cost data per se but can be used to identify costs per event by combining CPT codes with standard cost data. This approach decreases the internal validity of cost estimates for an individual site but greatly improves the validity of estimation of any resource use and cost differences between sites and has greater external validity of economic estimates made using the study costs [11]. The CPT codes in each visit record were used to assign a visit cost based on the median charge for the CPT code published for all medical practices in the United States in 2016 [12].

An exploratory data analysis was conducted to understand the breadth and specificity of the available data. Descriptive statistics, including measures of central tendency, were generated to identify outliers and to track data consistency for future downloads. This study provides the first descriptive analysis performed on the 2016 data as part of the development of a common data model for the study. The analysis was performed using SAS (version 9.4). Groups were compared using chi-square tests for categorical variables and 2-talied *t* tests (normally distributed) or Mann-Whitney *U* or Wilcoxon tests (nonnormally distributed) for continuous variables.

## Results

### Feasibility of Variable Downloads by Study Sites

The parent SIU study was launched in December 2017 at 10 clinical sites, with 2016 EHR data due on January 31, 2017. Site personnel often reported a lack of consistent access to information technology (IT) and data management specialists as the primary explanation for the delay in data submission. Although the sites varied in the length of time to obtain and prepare data (2-6 months), all sites successfully submitted data to the designated data repository by May 2018. The primary variables requested to monitor the cascade of care included the date of visit, age, sex, height, weight, race, ethnicity, VL, and ICD-10 codes for other diagnoses. In total, 70% (7/10) of the sites provided data for the primary variables via the extraction of electronic records from their EHR system. The remaining sites provided the primary variables using manual data extraction.

The process of data cleaning and transforming the data into a common data model format was an extremely labor-intensive process that required >400 hours of expert programming and data management work. More importantly, it required extensive consultation between individuals with informatics programming expertise and researchers with HIV-specific treatment data experience. The extent of the work required to transform the data into a common analyzable data model was unexpected. Some of the most time-consuming tasks were needed because the EHR systems for a site did not use clearly defined uniform values for many of the variables. Definitions for some demographic variables (sex, gender, race, and ethnicity) varied within a site’s data download owing to the hand entry of variables, and the use of upper and lower cases, codes, and narrative descriptions. Visit variables that contained ICD-10 diagnosis codes and CPT codes were often problematic because they were sometimes extracted as string variables, separated by a mix of spaces and semicolons. Medication files were especially labor-intensive because similar medications had different names, spellings, abbreviations, and patterns of upper cases and hyphens. Laboratory tests had different definitions of VL and CD4 cell counts, and the names of other laboratory tests varied greatly and were sometimes not clearly identified. These difficulties were resolved by querying sites for information and by using site-specific detailed cleaning programs written for this purpose. Laboratories have varying lowest detectable limits for VL; therefore, this study records undetectable VL as defined by each study site.

### Baseline Participant Characteristics

A total of 1093 patients were enrolled in 2016 (Table 1). The demographic variables had the lowest frequency of missing values (4%-5%). ARV medication records were not available for 17.75% (194/1093) of the patients. The VL was missing for 6.40% (70/1093) of the patients. This variable was hand-extracted at the site if missing, indicating that some patients did not have a VL record entered as structured data into their EHR system. A CD4 cell count, an important measure of immune status, was missing for approximately 28.81% (315/1093) of cases. However, we did not require this variable to be hand extracted, so it is possible that CD4 cell tests for many patients may have been performed in an external laboratory with results uploaded as a report. It may be important to make CD4 cell count required by hand extraction when electronic extraction is not possible, if we need to use this variable to measure changes in immune status over time.

**Table 1:** Characteristics of the cascade monitoring study population at baseline (N=1093).

Characteristic		Patient value	Number of patients for whom 2016 data were missing^a^
Age (years), mean (SD)		20.8 (2.5)	41
Height (cm), mean (SD)		169.9 (10.9)	104
Weight (kg), mean (SD)		76.8 (18.5)	84
BMI (kg/m^2^), mean (SD)		26.6 (6.6)	106
Male sex, n (%)		730 (70.67)	60
**Race, n (%)**			50
	Black	769 (73.73)	
	White	89 (8.53)	
	Hispanic	105 (10.07)	
	Other	80 (7.67)	
Antiretroviral medication, n (%)		884 (98.3)	194
Patients with AIDS, n (%)		77 (9.9)	315
**Site reported sample size, n (%)**			
	Baltimore, Maryland	94 (8.60)	N/A^b^
	Birmingham, Alabama	69 (6.31)	N/A
	Brooklyn, New York	115 (10.52)	N/A
	Los Angeles, California	85 (7.78)	N/A
	Miami, Florida	54 (4.94)	N/A
	Memphis, Tennessee	193 (17.66)	N/A
	Philadelphia, Pennsylvania	78 (7.14)	N/A
	San Diego, California	105 (9.61)	N/A
	Tampa, Florida	219 (20.03)	N/A
	Washington, District of Columbia	81 (7.41)	N/A
Mean total cost per visit (US $) across CPT^c^ codes, mean (SD)		325 (376)	N/A
CD4 Laboratory results, mean (SD)		609.4 (316)	315
Viral load laboratory results, mean (SD)		25,608 (114,184)	70
Visits, mean (SD)		5.4 (4.6)	24
Patients with laboratory visits >190 days apart in 2016, n (%)		84 (8.15)	N/A
Patients in 2016 with no record in 2017, n (%)		202 (18.48)	N/A

^a^Missing 2016 data elements for race, age, sex, height, and weight were extracted from 2017, if available.

^b^N/A: not applicable.

^c^CPT: Current Procedural Terminology.

### Patient Care Continuum Outcomes

Only 2 of the 4 CDC treatment cascade measures can be assessed using routine EHR data. EHR data are not adequate for monitoring HIV testing or linkage to care because the denominator data are not available. However, the data work well for measuring ART initiation and adequately for retention in care. The criterion used to indicate less-than-optimal retention visit patterns for CD4 or VL testing is detailed in the *Methods* section. On the basis of this criterion, approximately 82.97% (887/1069) of the patients met the minimum criteria for laboratory visit frequency. On average, our patient cohort had 5.4 (SD 4.6) laboratory visits per year in 2016, with a range of 1 to >150 visit records. All prescribed ARV and other medications were requested for the study participants. A total of 98.3% (884/899) of the patients from sites that were able to extract medication records had at least one record of prescribed ARV medication. This finding is in line with the Joint United Nations Program on HIV proposed target that 90% of all people with diagnosed HIV infection receive sustained ART by 2020 [13].

We compared patients with undetectable baseline VL to those with a VL value above the level of detection used by the VL test in their center (Table 2). Minority race and ethnicity patients were more likely to have detectable VL (*P*=.03) with 77.9% (313/402) of patients with detectable VL being of Black or African American race compared with 72.6% (355/489) of patients being Black or African American with undetectable VL. The findings showed poorer immune marker values for patients with a detectable VL. A total of 45.6% (194/424) of these patients had a mean VL of >10,000, and 29% (41/141) had a CD4 cell count that classified them as having AIDS. In addition, a small number of patients in the group with suppressed VL at baseline (7/521, 1.3%) also had a CD4 cell count that classified them as having AIDS.

The mean CD4 cell count was 619 (SD 310), indicating that many of the 945 patients with CD4 cell count values had a relatively good immune status. However, 5.7% (54/945) of patients with available CD4 cell count values had a baseline count of <200, which classified them as having AIDS by the CDC. The VL burden was reported to be undetectable in 55.1% (521/945) of the patients. However, a total of 20.5% (194/945) of all patients with a VL record had a baseline (first VL available in 2016) VL of >10,000 copies/mL.

**Table 2:** Characteristics of patients with undetectable and detectable VL^a^ at baseline (N=945).^b^

Characteristics			Patients with undetectable baseline VL		Patients with detectable baseline VL		*P* value	
Patients in the study, n (%)			521 (55.1)		424 (44.9)		.002	
Age (years), mean (SD)			20.8 (2.6)		20.8 (2.5)		.71	
Male sex, n (%)			328 (62.9)		296 (69.8)		.06	
**Race and ethnicity, n (%)**								.03
	Black	355 (68.1)		313 (73.8)				
	White	46 (8.8)		20 (4.7)				
	Hispanic	26 (5.0)		28 (6.6)				
	Other	62 (11.9)		41 (9.7)				
VL >10,000, n (%)			N/A^c^		194 (45.7)		—^d^	
Log_10_ VL if VL was detectable, mean (SD)			N/A		3.6 (1.2)		—	
CD4 cell count, mean (SD)			707 (274)		512 (321)		<.001	
CD4 count <200 or with AIDS, n (%)			7 (1.3)		47 (11.1)		<.001	

^a^VL: viral load.

^b^A total of 148 patients had no baseline VL measures.

^c^N/A: not applicable.

^d^Statistical testing was not performed.

## Discussion

### Principal Findings

In preparation for the study, it became clear that some sites that were considered for inclusion were unable to provide specific variables. Therefore, the study team identified 9 mandatory variables for which information must be provided for a site to remain in the study. These mandatory variables (needed to monitor the treatment cascade) included the following: (1) VL, (2) date of visit, (3) age, (4) weight, (5) height, (6) ICD-10 and CPT codes, (7) sex, (8) race, and (9) ethnicity. For the most part, the sites were able to provide information regarding the required data. The use of EHR data is effective in assessing the patterns of completed appointments. However, in most cases, these data are insufficient for identifying patterns of missed appointments because such misses are not captured in the EHR system. Direct electronic download may not be possible in all situations; however, our data management team was able to work with the sites to develop a successful plan for data abstraction. Sites with good access to data management resources can work more efficiently for CM purposes. However, we do not know the IT resources of the study sites. Many clinic site personnel shared with the study investigators that they were not sure who to contact for help or how much help they could expect from their IT service group. Clinical staff members at many sites have little day-to-day contact with IT data specialists. This is an important issue that should be part of the selection process when looking for site partners for clinical studies that plan to use EHR data. Study planners should determine the feasibility of additional funding for organizations in need of additional IT or data management resources because these needs are not always obvious at the planning stage.

A number of barriers to EHR use and lessons learned as part of the CM study have come to light. First, the required variables that are *visible* to clinicians on the EHR user interface may not be readily available for electronic download. A number of important variables are part of the narrative text or are simply not recorded as structured data. VL, height, and weight are examples of information contained within clinic notes or PDF files of the results of tests performed off-site by a vendor and only available as scanned reports. An HIV surveillance study found that differences in estimated care engagement and viral suppression between data sources revealed incomplete laboratory reporting and that patients received care from multiple providers [14]. Such findings highlight the potential unavailability of information pertinent to treatment CM and make monitoring *dashboard* construction infeasible. Some data require *hand extraction*, and sites cannot easily find the data. Patient height is often not recorded, which makes it difficult to define important measures, such as overweight, obesity, or BMI. Laboratory data extracts may lack important definitions of *normal*. Undetectable VL is especially difficult to standardize in a common data model because the laboratory definition varies according to the type of test used. Some important characteristics of the youth population infected with HIV were not recorded. Sexual partner preference or sexual orientation is not routinely documented in clinics. Sex is sometimes provided as gender and sometimes as biological sex. Missing data are common, and there are few explanations for missing data, but our comparison of data from 2016 and 2017 indicates that variables with missing values in 1 year tend to be within a normal range if present subsequently, so we suspect that they are most likely missing at random. This means that current statistical approaches for dealing with missing data may not be good choices for use in EHR data studies. In addition, there may be a discrepancy between the information provided during a consultation and that reported in the EHR [15], and vital information regarding participation is not captured or readily accessible. Much important information in an EHR for youth infected with HIV is located in unstructured data such as physician, nurse, or social worker notes. Neither data formatting nor hand cleaning will solve this lack of structured data. What may be needed is text recognition software and natural language processing approaches. However, these methods are costly and pose problems with regard to deidentifying data.

The study findings indicate that a substantial disease burden is present in this very young population infected with HIV. However, EHR data can be systematically obtained from multiple clinical sites and used to meaningfully capture the CDC’s continuum of care measures. In addition, this study used standard cost values attached to CPT codes to estimate the cost per visit. This approach delineates the differences in prices between sites and proves the internal and external validity of cost estimates. To the best of our knowledge, this is the first EHR study to use this approach.

As previously stated, to fully benefit from advances in HIV treatment, youth must actively engage in each step of the treatment cascade. Although EHRs sufficiently capture pathology and many other aspects of health, obtaining meaningful indicators of health and treatment outcomes regarding patient activity measures of the care continuum (eg, keeping appointments and taking ART medications as prescribed) proved more difficult. Among other recommendations, Newman-Griffis et al [16] suggested specific actions to improve the capture and analysis of activity and participation information throughout the continuum of care, including (1) making activity and participation annotation standards and data sets available to the broader research community and (2) establishing standards for how and when to document activity and participation status during clinical encounters. A data-driven approach leveraging current techniques in health informatics to extract information about function, particularly activity and participation, is needed [16,17].

### Conclusions

The work performed as part of the CM study has greatly advanced our understanding of the strengths and weaknesses of using EHR data to monitor the treatment cascade in youth infected with HIV. The analysis of the data from the first year of the study indicates that EHR data can be extracted from diverse sites and converted into analyzable data sets capable of monitoring important variables in the HIV treatment cascade. However, the success of any such effort will depend entirely on a solid collaboration between investigators and staff at the sites and the study team responsible for the cleaning and standardization of the data. The support of clinical investigators at the site has been essential for the success of the project, and the study would not have been possible without the extraordinary level of commitment and willingness to overcome obstacles exhibited by the clinical site staff. The work effort was much greater than expected for the CM study team. However, it appears that the careful programming work performed to transform the 2016 data is paying off because the 2017 data have been downloaded, and early cleaning efforts indicate that much of the work conducted for 2016 is usable for the 2017 data download. This is encouraging and indicates that *up-front* work on a standard data model pays off in terms of efficiency in subsequent years. This study provides pilot data on the use of EHRs to determine who and when youth living with HIV/AIDS disengage from the cascade and a broad, nonintrusive, and efficient way of assessing whether SIU interventions improve cascade outcomes. We conclude that although the use of EHR data for treatment CM for youth with HIV is labor-intensive and not ideal for some measures, it works for much of what we need to know about monitoring retention in care. Thus, it has the potential to become an essential tool for measuring the achievement of the goal of improving access to quality care for youth infected with HIV in the United States.

### Future Directions

The longitudinal design of this study will allow for the calculation of cascade measures (ART prescription, viral suppression, and retention in care) throughout the study time fame of 2016 to 2021. In addition, advanced analytic procedures will be used to model care retention based on both patient-related and clinical characteristics, resulting in the creation of patient phenotypes for youth living with HIV/AIDS. Creation of the phenotypes will facilitate the identification of relevant predictors associated with dropout at any stage of the cascade and will be used to estimate the cost per quality-adjusted life year expected from cascade outcomes. As part of the identification of the larger cost-of-illness burden of cascade lapses for youth living with HIV/AIDS, we will use the EHR phenotypes and archival data from Medicaid or privately insured populations to model the extent of cascade interruptions present in other practice settings. The data will be combined with the individual cost weights to estimate the variations in the economic burden that cascade disruptions for youth living with HIV/AIDS place on US communities.

With the development of phenotypes, we will be able to extend our study findings to identify lapses in the treatment cascade for youth living with HIV/AIDS using large national databases. This process will permit the enumeration of these patient phenotypes nationwide and the description of the related annual economic costs of care. This will enable us to identify meaningful variations between communities that can then serve as the basis for targeted interventions.

## Appendix

Multimedia Appendix 1 List of study variables.

Multimedia Appendix 2 Summary of Centers for Disease Control and Prevention care continuum measures.

## Abbreviations

ART: antiretroviral therapy
ARV: antiretroviral
ATN: Adolescent Trials Network for HIV/AIDS Interventions
CDC: Centers for Disease Control and Prevention
CM: cascade monitoring
CPT: Current Procedural Terminology
EHR: electronic health record
ICD-10: International Statistical Classification of Diseases and Related Health Problems–10th Revision
IT: information technology
SIU: Scale It Up
VL: viral load

## References

[1] Fauci AS, Redfield RR, Sigounas G, Weahkee MD, Giroir BP (2019). Ending the HIV epidemic: a plan for the United States. JAMA.

[2] (2018). National Center for HIV, Viral Hepatitis, STD, and TB Prevention. Centers for Disease Control and Prevention.

[3] Guilamo-Ramos V, Thimm-Kaiser M, Benzekri A, Futterman D (2019). Shifting the paradigm in HIV prevention and treatment service delivery toward differentiated care for youth. NAM Perspectives.

[4] (2019). Understanding the HIV care continuum. Centers for Disease Control and Prevention.

[5] Kay ES, Batey DS, Mugavero MJ (2016). The HIV treatment cascade and care continuum: updates, goals, and recommendations for the future. AIDS Res Ther.

[6] Mugavero MJ, Amico KR, Horn T, Thompson MA (2013). The state of engagement in HIV care in the United States: from cascade to continuum to control. Clin Infect Dis.

[7] Lally MA, van den Berg JJ, Westfall AO, Rudy BJ, Hosek SG, Fortenberry JD, Monte D, Tanney MR, McFarland EJ, Xu J, Kapogiannis BG, Wilson CM, Adolescent Medicine Trials Network for HIV/AIDS Interventions (ATN) (2018). HIV continuum of care for youth in the United States. J Acquir Immune Defic Syndr.

[8] Pennar AL, Dark T, Simpson KN, Gurung S, Cain D, Fan C, Parsons JT, Naar S (2019). Cascade monitoring in multidisciplinary adolescent HIV care settings: protocol for utilizing electronic health records. JMIR Res Protoc.

[9] Zanoni BC, Mayer KH (2014). The adolescent and young adult HIV cascade of care in the United States: exaggerated health disparities. AIDS Patient Care STDS.

[10] (2019). Understanding the HIV care continuum. Centers for Disease Control and Prevention.

[11] Simpson KN, Tilley BC (2012). Economic analysis of secondary trial data. Prog Cardiovasc Dis.

[12] Davis JB (2016). Medical fees U.S. 2016.

[13] (2017). 90-90-90 - an ambitious treatment target to help end the AIDS epidemic. UNAIDS.

[14] Arey AL, Cassidy-Stewart H, Kurowski PL, Hitt JC, Flynn CP (2019). Evaluating HIV surveillance completeness along the continuum of care: supplementing surveillance with health center data to increase HIV data to care efficiency. J Acquir Immune Defic Syndr.

[15] Lacroix-Hugues V, Azincot-Belhassen S, Staccini P, Darmon D (2019). Differences between what is said during the consultation and what is recorded in the Electronic Health Record. Stud Health Technol Inform.

[16] Newman-Griffis D, Porcino J, Zirikly A, Thieu T, Camacho Maldonado J, Ho PS, Ding M, Chan L, Rasch E (2019). Broadening horizons: the case for capturing function and the role of health informatics in its use. BMC Public Health.

[17] Beard JR, Officer A, de Carvalho IA, Sadana R, Pot AM, Michel JP, Lloyd-Sherlock P, Epping-Jordan JE, Peeters GM, Mahanani WR, Thiyagarajan JA, Chatterji S (2016). The World report on ageing and health: a policy framework for healthy ageing. Lancet.

