# Supplementary material for: Feasibility of Using Electronic Health Records for Cascade Monitoring and Cost Estimates in Implementation Science Studies in the Adolescent Trials Network for HIV/AIDS Interventions
Source: JMIR Form Res. 2022 Apr 25;6(4):e25483. doi: 10.2196/25483 (PMC9086886; doi:10.2196/25483)
Supplement: Multimedia Appendix 1 [file formative_v6i4e25483_app1.docx]

Appendix

Time invariant variables requested

- Patient ID: non-PHI
- Current Age
- Age at HIV Diagnosis
- Date of HIV Diagnosis
- Gender
- Race
- Ethnicity
- Sexual-Orientation
- Mode of Transmission
- Date of Entry into Care
- Date of Death (if applicable)

Time Variant Variables requested

- Patient ID (invariant but required in this file)
- Visit Date
- Antiretroviral Medications – Name
- Other non-HIV medication - Name
- Viral load results
- CD4 cell count result
- Height
- Weight
- Blood pressure
- Cholesterol Panel
- Blood glucose
- Tobacco use
- STI test results
- Patient Zip Code
- ICD-10 Diagnosis Codes
- CPT-4 Procedure Codes
- HCPCS Procedure Codes
