# Supplementary material for: Feasibility of Using Electronic Health Records for Cascade Monitoring and Cost Estimates in Implementation Science Studies in the Adolescent Trials Network for HIV/AIDS Interventions
Source: JMIR Form Res. 2022 Apr 25;6(4):e25483. doi: 10.2196/25483 (PMC9086886; doi:10.2196/25483)
Supplement: Multimedia Appendix 2 [file formative_v6i4e25483_app2.docx]

Appendix 2.

Summary of CDC Continuum Steps

1. Diagnosed: Measures the percentage of the total number of people living with HIV whose infection has been diagnoses
2. Receipt of Care: Measures the percentage of persons with diagnosed HIV who had at least one CD4 or viral load test
3. Retained in Care: Measures the percentage of persons with diagnosed HIV who had two or more CD4 or viral load test performed at least 3 months apart
4. Viral Suppression: A viral load test result of <200 copies/mL at the most recent viral load test during measurement year.
5. Linked to care: Measures the percentage of people receiving a diagnosis of HIV in a given calendar year who had one or more documented CD4 or viral load tests within 30 days of diagnosis

<https://www.cdc.gov/hiv/pdf/library/factsheets/cdc-hiv-care-continuum.pdf>
